# High protein diet increases the risk of allergic sensitization but not asthma in mice through modulation of the cytokine milieu toward Th2 bias^☆^

**DOI:** 10.1016/j.waojou.2025.101031

**Published:** 2025-02-06

**Authors:** Waleed Al-Herz, Fawaz Azizieh, Raj Raghupathy

**Affiliations:** a Department of Pediatrics, College of Medicine, Kuwait University, AND Allergy and Clinical Immunology Unit, Pediatric Department, Al-Sabah Hospital, Kuwait City, Kuwait; b Department of Mathematics and Natural Sciences, Gulf University for Science and Technology, And College of Integrative Studies, Abdullah Al Salem University, Kuwait; c Department of Microbiology, College of Medicine, Kuwait University, Kuwait City, Kuwait

**Keywords:** Diet, High-protein, Immunoglobulin E, Cytokines, Hypersensitivity, Ovalbumin, Asthma

## Abstract

**Introduction:**

The role of different nutrients in allergic sensitization is not clear. In this study we aimed to determine the effect of high protein (HP) diet on allergic sensitization, cytokine profile, and asthma in mice.

**Methods:**

Seven- to eight-week old female BALB/c mice were fed either normal (ND) or HP diet and were sensitized with ovalbumin intraperitoneally followed by intranasal challenge. Allergic sensitization was tested by measuring anti-ovalbumin (OVA) IgE, IgG1, and IgG2a antibodies. Cytokine levels were tested by multiplex ELISA in splenocyte supernatants after stimulation. Airway inflammation was tested by measuring total and differential cell counts in bronchoalveolar lavage fluid and by measuring bronchial mucus production, goblet cell hyperplasia and perivascular and peribronchial inflammation severity scores by histologic examination.

**Results:**

Mice fed HP diet had a significant increase in weight and higher levels of OVA-specific IgE and IgG1 antibodies compared to the ND group (P-values 0.002, 0.007 and <0.001, respectively). In addition, they showed a selective Th2 bias in cultured splenocyte supernatants compared to the ND group as demonstrated by higher IL-4 and IL-6 levels (P-values <0.001 and 0.011, respectively) and higher ratios of Th2 to Th1 cytokines. However, the level of airway inflammation was comparable between both groups.

**Conclusions:**

HP diet increases the risk of allergic sensitization though increase in Th2 cytokines. Efforts should be made to define the upper limit of protein in the diet that does not predispose to allergic sensitization. The effect of diet on health should remain a focus of research for the establishment of optimal health and resilience.

## Introduction

There has been a dramatic rise in the prevalence of atopic diseases in both developed and developing countries, especially in children. The Phase III International Study of Asthma and Allergies in Childhood (ISAAC) reported worldwide increases in the prevalence of atopic disorders compared to the prevalence reported in the Phase I study.^1^ Atopic diseases cause significant morbidity, mortality, and socio-economic burdens.^2^ For example, the economic costs of asthma in the United States in 2007 in terms of direct and indirect costs was $ 19.7 billion US dollars.^3^ A study from the European Union showed that the costs for patient insufficiently treated for allergy range between € 55 and € 151 billion Euros per annum due to absenteeism and presenteeism.^4^

Atopic diseases are heterogeneous and involve important gene-environmental interactions. In children the manifestations may occur in a characteristic sequence known as the atopic or allergic march.^5^ Although atopic march is highly controversial and only a minority of patients follow this march,^6^ the ﬁrst signs of atopic diseases are usually food allergy and atopic dermatitis. As these signs of atopic disease are often observed in the ﬁrst 3 years of life, they are thought to be related to the maturation of the immune system.^6^

The 2 major subsets of CD4+ T helper cells, Th1 and Th2, have different patterns of cytokine production and different roles in immune responses. Th2 cells are involved in the initial steps of the development of allergies through the secretion of Th2 cytokines which promote B cells isotype switch to IgE.^7^ In addition, Th2 cells regulate mast cell function and homeostasis and stimulate eosinophil activation and recruitment to tissue sites.^8^^,^^9^ More recently, the role of Th17 cells and its proinflammatory cytokine IL17 in allergic diseases has become more evident.^10^

The role of nutrition in asthma prevention and treatment was recently reviewed with some evidence suggesting that the consumption of plant-based foods and reduced consumption of animal products might protect against asthma development and improve asthma symptoms.^11^ However, firm dietary recommendations to prevent atopic diseases have been limited to exclusive breast feeding for the first 3–4 months, and early introduction of complementary food between the ages of 4–6 months.^12^ There is some evidence that overweight/obesity in women before and during pregnancy as well as in children and adolescents promote the development asthma.^13^ A recent report from the American Academy of Pediatrics (AAP) states that there is a lack of evidence that partially or extensively hydrolyzed formula prevents atopic disease in infants and children, even in those at high risk for allergic disease.^12^

A previous study showed that mice fed a low protein diet resulted in the induction of oral tolerance against ovalbumin (OVA) through down regulation of IL-4.^14^ In this study we aimed to determine the effect of HP diet on allergic sensitization, the cytokines profile and asthma in mice. The rationale of this study is that diet and nutrition are modifiable variables; therefore, a better understanding of their effects on allergic sensitization is critical.

## Materials and methods

### Animals and diets

Seven-to eight-week-old female BALB/c mice were used for the experiments. The mice had free access to food and water. Animal foods (normal and HP) were purchased from Research Diets, Inc (NJ, USA); food pellets consisted of purified ingredients and were ovalbumin-free. The details of the diets and the composition are shown in Table 1.

**Table 1:** Details of the normal and high protein diet

**Diet**	**Normal**	**High Protein Diet**		**Normal**	**High Protein Diet**
**Ingredient (gm)**			**Total (gm)**	**1047.35**	**1073.95**
Casein	220	440			
L-Cystine	3.3	6.6	**Gm**		
			Protein	196.9	393.8
Corn starch	470.2	273.5	Carbohydrate	674.0	477.3
Maltodextrin 10	125	125	Fat	53.0	53.0
Sucrose	68.8	68.8	Fiber	50.0	50.0
Cellulose	50	50	**gm%**		
			Protein	18.8	36.7
Lard	0	0	Carbohydrate	5.1	4.7
Soybean oil	53	53	Fat	5.1	4.9
			Fiber	4.8	4.7
Mineral mix S10026	10	10			
Dicalcium phosphate	13	13	***Kcals***		
Calcium carbonate	5.5	5.5	Protein	788	1575
Potassium citrate, 1H2O	16.5	16.5	Carbohydrate	2696	1909
			Fat	477	477
Vitamin mix V10001	10	10	Total	3961	3961
Choline bitartrate	2	2			
			***kcal%***		
Red dye #40,	0	0.025	Protein	20	40
Blue dye #1,	0.01	0.025	Carbohydrate	68	48
Yellow dye #5,	0.04	0	Fat	12	12

### Study protocol

Mice were divided into 2 groups (n = 12 in each group) based on the type of diet, either normal (ND) or high protein (HP).

**Fig. 1:**
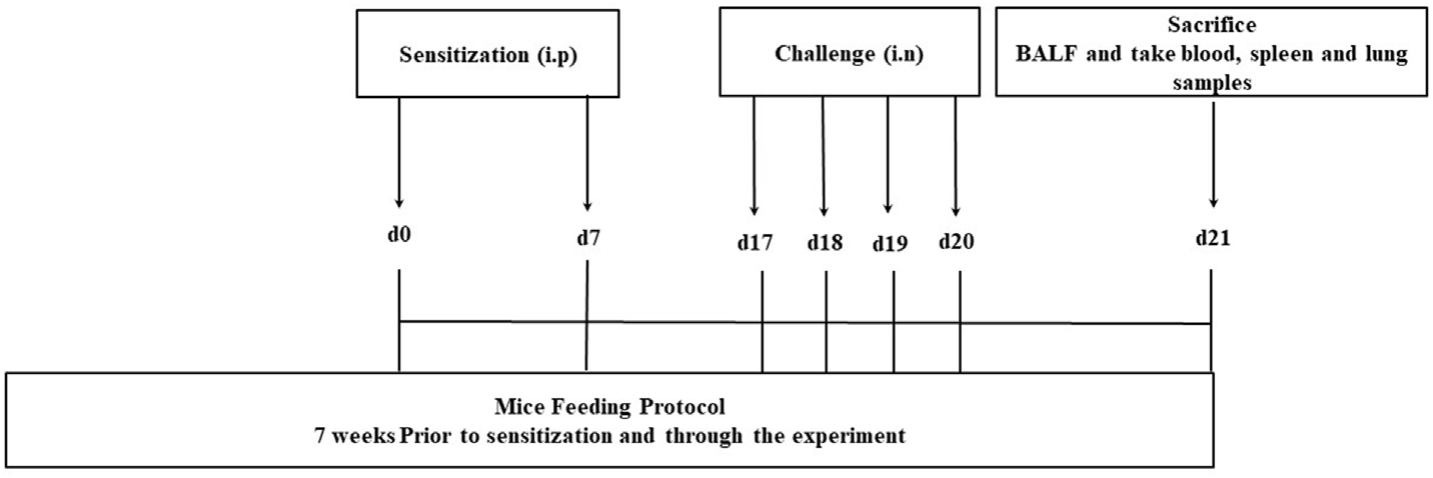
Schematic illustration of the study protocol. i.p: intraperitoneal, i.n: intranasal, BALF: bronchoalveolar lavage fluid.

The mice were sensitized with OVA 7 weeks after feeding them either diet and was followed by intranasal challenge. All mice were sacrificed with an overdose of halothane at the end of the study protocol. Bronchoalveolar lavage fluid (BALF), lung tissue, blood, and spleen were collected from all the mice (Fig. 1).

### Sensitization of mice with ovalbumin and intranasal challenge

Sensitization with OVA was done as described previously^15^ by intraperitoneal injection with 100 μg OVA in 0.2 ml of Alum Adjuvant (Thermofisher catalog #771616). This was repeated 7 days later to boost the sensitization. Mice were challenged intranasally, once a day, over 4 consecutive days, with 30 μg OVA in 50 μl PBS on days 17–20.

### Serum levels of OVA specific IgE, IgG1, and IgG2a

Levels of anti-OVA IgE, IgG1, and IgG2a antibodies were measured in sera using a homemade sandwich ELISA as described previously.^16^ OVA was biotinylated using a kit (Pierce) and used for detection of antibodies. Briefly, microtiter ELISA plates were coated overnight with rat anti-mouse IgE (Becton Dickinson #553413) or rat anti-mouse IgG1 (Becton Dickinson # 553440) or rat anti-mouse IgG2a antibodies (Becton Dickinson BD # 553387). After washing the wells, blocking buffer (0.5% gelatin in wash buffer or 3% Rat serum in wash buffer) was added and the plates incubated for 2 h. Appropriate reference standard OVA-IgE (Chondrex #3006) or OVA-IgG1 (Chondrex # 7093) or OVA-IgG2a (Chondrex # 7095) and diluted serum samples were then added to the wells and incubated for 3 h. This was followed by incubation with OVA-Biotin for 1 h and with streptavidin-HRP for 30 min. Tetramethylbenzidine substrate was added and the enzyme-substrate reaction stopped after 15 min, and the optical density measured at 450 nm.

### *In vitro* stimulation of splenocytes

Splenocyte stimulation was done as described previously.^17^ Wells of tissue culture microtiter plates were aliquoted with 150 μl of cells at 5 × 10^5^ cells/well in RPMI-1640 medium and stimulated with 50 **μ**l of OVA at a concentration of 20 μg/well. Stimulation with the antigen was done in triplicate wells. The incubation was for 96 h. At the end of the stimulation, supernatants were collected and stored at −80 °C until assayed for cytokines.

### Cytokine secretion by splenocytes

A multiplex ELISA-based immunoassay, containing dyed microspheres conjugated with monoclonal antibodies specific for target cytokines, was used according to the manufacturer's instructions (Merck Millipore, Darmstadt, Germany). Soluble cytokines were measured using mouse cytokine commercially available kits for 5-plex panel (IFN-γ, TNF-α, IL-6, IL-17, and IL-4) (MCYTOMAG-70K). Concentrations of all analytes were determined using a MAGPIX array reader (Luminex, Austin, TX) that quantitates multiplex immunoassays in a 96-well format with very small sample volumes. Analyte concentrations were calculated using standard curves, with software provided by the manufacturer (Luminex Manager Software). The sensitivity of each of the assays in pg/mL was as follows TNF-α: 2.3, IL-6: 1.1, IL-17: 0.5, IL-4: 0.4., and IFN-γ: 1.1. Quality control measures were followed as per the recommendation of the manufacturers. The range of intra-assay and inter-assay coefficients of variation were 1.7–2.6% and 4.1–15% respectively. Accuracy in terms of recovery in matrix ranged from 84.9 to 96.7%.

### Bronchoalveolar lavage fluid (BALF) cell counts and lung histology

BALF testing and lung histologic examination were done as described previously.^15^ BALF results are expressed as total cell count/ml and as total macrophages, lymphocytes, neutrophils, and eosinophils/ml. The number of BALF cells was calculated using a particle-size counter (Z1 Single Threshold; Beckman Coulter) then, cytosmears were prepared, and cells were stained with Diff-Quik. Afterwards, a differential count of 200 cells was performed using standard morphologic criteria. The total cell count reflects the number of cells per mL of BAL fluid. The differential cell count represents the absolute number for each cell type/mL of BAL.

Lung histological sections were processed and examined under light microscopy after staining separately with hematoxylin and eosin (H&E) and periodic acid–Schiff (PAS). The severity of perivascular and peribronchial inflammation, mucus intensity and goblet cell hyperplasia were scored independently by an experienced histologist unfamiliar with the slides and specific diet intervention to eliminate bias. Score coding was as follows: 1 = normal, 2 = mild, 3 = moderate, 4 = severe and 5 = highly severe.

### Statistical analysis

Statistical analyses were performed using the SAS 9.4 software (SAS Institute, Cary, NC, USA). The statistical significance level was set at α = 0.05 for all the association analyses. Normality of distribution of continuous variables was assessed using the Shapiro-Wilk test, and homogeneity of variance (uniform variance) was evaluated by Levene's test (Supplemental Table). Variables that demonstrated normal distribution and uniform variances (increase in weight, Ova-IgE, Ova-IgG1, IL-4, IL-6, total cell count, absolute eosinophil count, absolute neutrophil count, absolute macrophage count, and absolute lymphocyte counts) were described by estimating their mean and 95% confidence interval (CI). Variables that violated the normality of distribution and/or the uniform variances (Ova-IgG2a, IL-17, IFN-γ, TNF-α, mucus integrity score, and cellular infiltration score) were described by estimating their median and interquartile range (IQR). T-test was used to evaluate associations between the exposure variable (diet type: ND diet vs HP diet) and biomarkers that had normal distribution and had uniform variance. The Wilcoxon rank sum test was applied to assess associations between the exposure variable (diet type: ND vs HP diet) and biomarkers that violated the normality of distribution and/or the uniform variances assumptions.

## Results

### Effect of different diets on weight

**Fig. 2:**
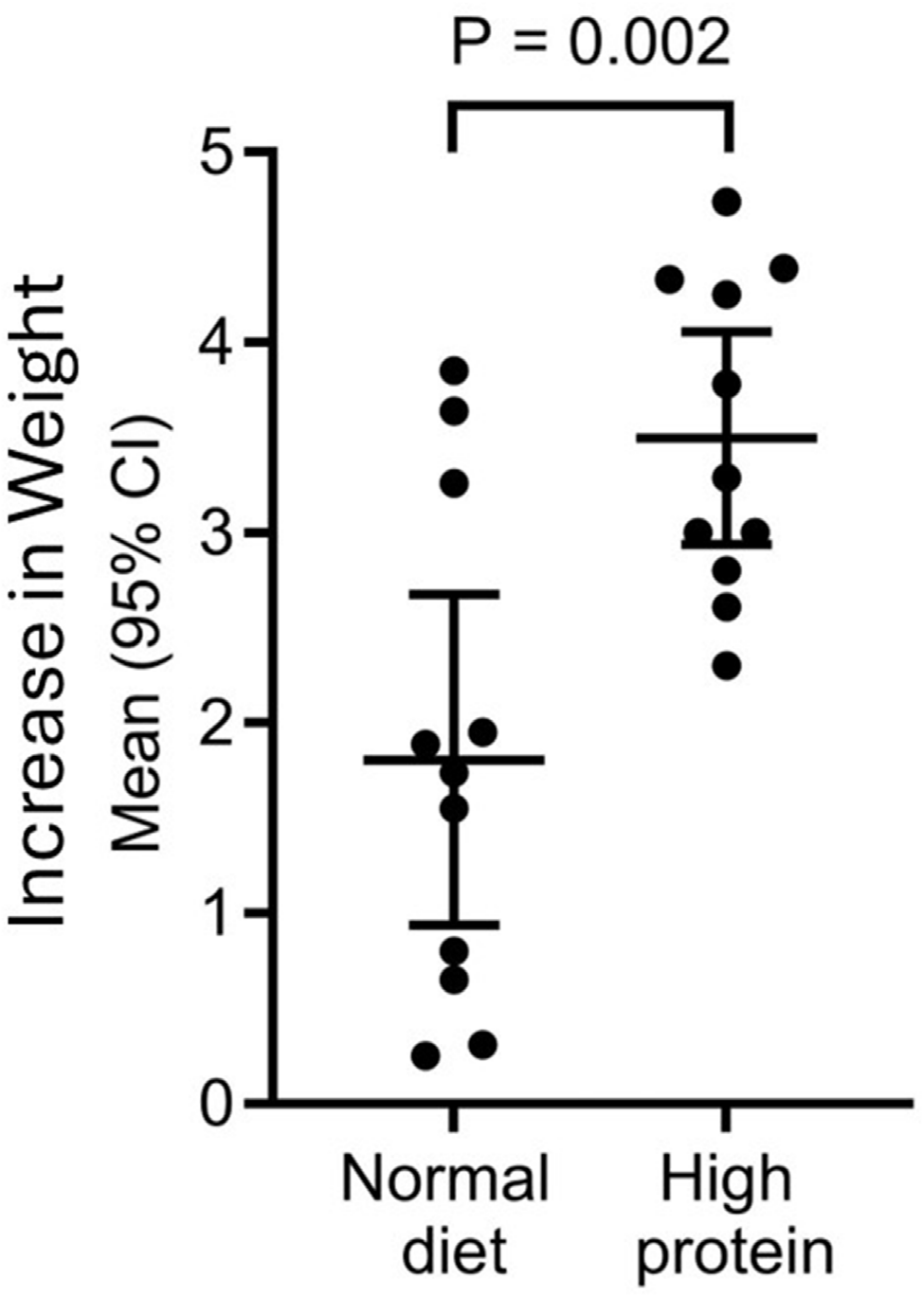
Increase in weight of mice after 7 weeks on either normal diet or high protein diet (n = 11 in each group). Mice fed high protein diet had significant increase in weight compared to the normal diet group. ns: not significant, CI: confidence interval.

Mice fed HP diet had a significant increase in weight at the end of the study compared to those fed ND shown in Fig. 2.

### Effect of different diets on sera levels of OVA-specific IgE, IgG1, and IgG2a

**Fig. 3:**
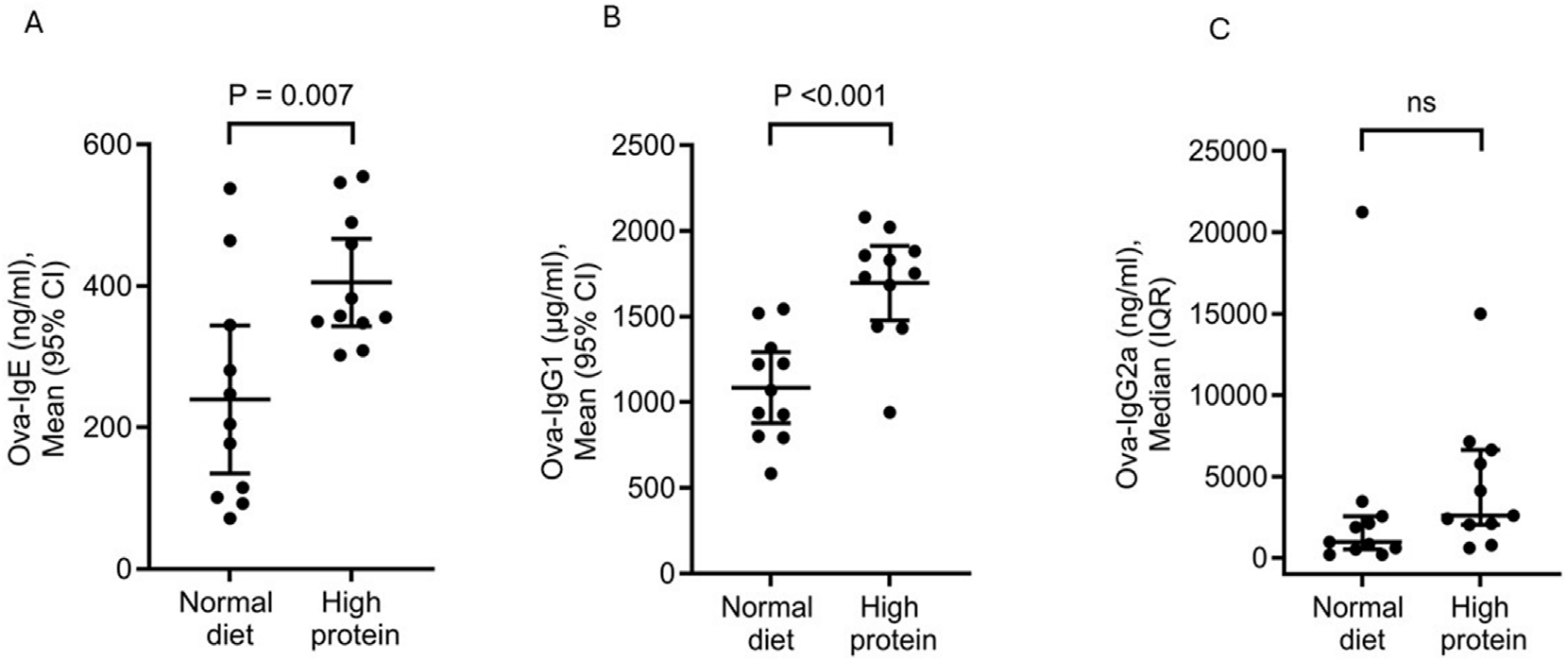
Effect of feeding mice for 7 weeks either normal diet or high protein diet on serum levels of (A) OVA-specific IgE (B) OVA-specific IgG1 and (C) OVA-specific IgG2a from OVA-sensitized mice (n = 11 in each group) after OVA intranasal challenge. The high protein diet group showed higher levels of both OVA-specific IgE and IgG1compared to the normal diet group. ns: not significant, CI: confidence interval. IQR: interquartile range.

Mice in the HP diet had significantly higher levels of OVA-specific IgE and IgG1 antibodies compared to those in the ND group shown in Fig. 3. In addition, levels of OVA-specific IgG2a antibodies were also higher in the HP group and approached statistical significance (P-value = 0.085).

### Effect of diets on levels of Th1, Th2 and Th17 cytokines in splenocyte culture supernatants

Table 2 shows the effect of diet on cytokine levels. Mice fed HP diet had higher IL-4, IL-6, TNF-α, and IL-17 in splenocyte culture supernatants compared to ND group. Interestingly, the level of IFN-γ was comparable between both diet groups.

**Table 2:** Cytokines levels in splenocyte culture supernatants.

Cytokine	Normal Diet	High Protein Diet	P-value
IL-4 (pg/ml)^a^	4.86 (3.01–6.72)	11.94 (9.00–14.87)	**<0.001**
IL-6 (pg/ml)^a^	63.66 (24.43–102.88)	142.56 (94.18–190.94)	**0.011**
IL-17 (pg/ml)^b^	1.39 (1.13–1.51)	1.64 (1.43–2.53)	**0.026**
IFN-γ (pg/ml)^b^	1.10 (1.10–3.51)	1.49 (1.10–2.51)	0.678
TNF-α (pg/ml)^b^	3.80 (3.20–4.62)	5.79 (5.09–7.17)	**0.012**

a Mean (95% confidence interval).

b Median (interquartile range 25th −75th percentiles).

To determine the specific Th type bias or possible dominance of 1 cytokine over another, we calculated the medians of ratios of Th2 to Th1 and Th2 to Th17 cytokines (Table 3). Overall, the ratios indicate a selective Th2 bias in the HP diet group compared to the ND group as evident by the statistically significantly higher IL-4:IFN-γ, IL-4:TNF-α, IL-6:IFN-γ, and IL-4:IL-17 ratios.

**Table 3:** Cytokines ratios in splenocyte culture supernatants.

	Normal Diet	High Protein	P-value
aIL-4:IFN-γ	3.25 (1.15–4.73)	5.51 (4.76–11.91)	**0.010**
aIL-4:TNF-α	1.13 (0.82–1.47)	2.15 (1.14–2.72)	**0.042**
aIL-6:IFN-γ	36.66 (19.06–51.87)	66.13 (45.38–106.17)	**0.021**
aIL-6:TNF-α	8.54 (5.80–29.74)	24.04 (17.16–31.76)	0.091
aIL-4:IL-17	3.25 (3.00–3.64)	6.72 (4.03–9.07)	**0.014**
aIL-6:IL-17	28.77 (17.09–94.17)	71.25 (39.60–93.59)	0.071

a Median of Ratios (IQR).

### Effect of diet on bronchoalveolar lavage fluid cell counts and lung histology

**Fig. 4:**
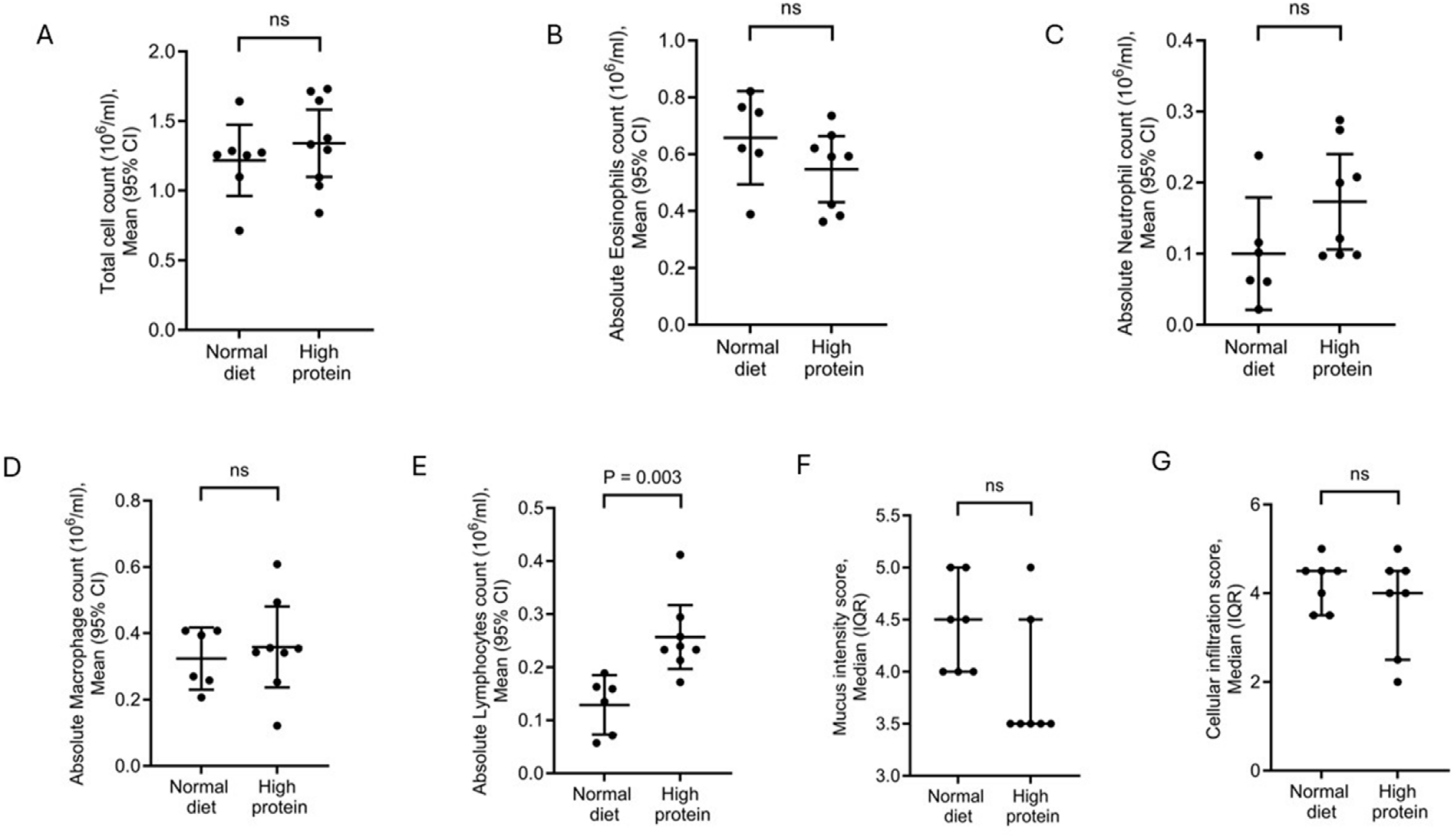
Effect of feeding mice for 7 weeks either normal or high protein diet on airway inflammation in OVA-sensitized mice after intranasal OVA challenge. (A–E) total and differential cell count in BALF (n = 6–8). The total cell count, macrophage, neutrophils, and eosinophil counts were comparable between both diet groups. However, mice in the high protein diet group had increased lymphocyte counts compared to the normal diet group. (F–G) Bronchoalveolar lavage fluid mucus intensity and lung cellular infiltration (n = 7 in each group). The scores were comparable between the 2 diet groups. ns: not significant. CI: confidence interval. IQR: interquartile range.

There was no statistically significant difference in the total cell, absolute macrophage, neutrophil, or eosinophil counts in BALF between mice fed ND or HP diet after intranasal challenge with OVA shown in Fig. 4A–D. However, there was a statistically significant increase in lymphocyte counts in mice fed HP diet compared to the ND group shown in Fig. 4E.

There were no differences between ND and HP diet groups after OVA intranasal challenge regarding bronchial mucus production and goblet cell hyperplasia and the perivascular and peribronchial inflammation severity scores shown in Fig. 4F–G.

## Discussion

In this study we observed that HP diet increases the risk of IgE-mediated sensitization through modulation of the cytokine milieu toward Th2 bias. A possible mechanism by which this occurs is by increased proliferation and function of type 2 innate lymphoid cells (ILC2). These cells are known for their importance in mediating type 2 immunity. Two recent studies have shown that both low protein diet and inhibitors of amino acid metabolism result in poor proliferation and reduced capacity to produce Th2 cytokines.^18^^,^^19^ Accordingly, we postulate that mice fed HP diet will have an exaggerated proliferation and function of ILC2 cells.

Another possible way that HP diet could increase the risk of allergic sensitization is by exerting an inhibitory effect on CD4^+^CD25+Foxp3 regulatory T cell (Treg) which play a crucial role in immunologic tolerance. A recent study on mice with chronic kidney disease that were fed low protein and high fiber diet showed a 1.7-fold and 2.4-fold increase in the number of Treg cells in spleen and in peripheral blood respectively, through modulation of the gut microbiome.^20^ However, it is unclear whether this effect was because of the low protein or high fiber in the diet. Another recent study showed that mice fed casein-gluten-soy diet had lower number of Treg cells in the small intestine epithelium compared to mice fed protein antigen-free solid diet containing free amino acids.^21^ Hence, we hypothesize that HP diet may have led to allergic sensitization by decreasing the number of Treg cells.

Diet is an important factor in determining the composition of gut microbiota,^22^ and several studies have reported the modulation of such composition by HP diet.^23^, ^24^, ^25^ One of these studies showed a link between dietary protein and gut microbial extracellular vesicles release and host secretory IgA response, which was associated with elevated expression of the transporter pIgR that was correlated with the highest expression of IL-4, a cytokine known to be involved in allergic sensitization. Other studies have shown a clear relationship between alterations in the gut microbiota and allergic disorders.^26^^,^^27^ We hypothesize that HP diet in this study may have caused modification in the gut microbiota that favors allergic sensitization.

In addition to the high level of OVA-specific IgE in mice fed HP diet, we showed that this group had significantly higher level of OVA-specific IgG1 compared to the ND group. IgG1 antibodies bind to FcγRIII on macrophages, basophils, and neutrophils and exert its biological effect during an allergic reaction due to platelet-activating factor and not histamine.^28^^,^^29^

Cytokine networks are among the most complex networks studied in biology; cytokines interact with one another via their signal transduction pathways with effects ranging from synergy to inhibitory actions. These cytokine connections and balances determine the nature and extent of the immune response as well as its biological consequences. Because relative levels of specific cytokines are probably of greater relevance than their absolute levels, we calculated the ratios of Th2 cytokines to both Th1 and Th17 cytokines. These suggest that a HP diet promotes a Th2 environment with its important role in allergic sensitization.

Our study showed that HP diet resulted in significant weight gain compared to ND diet. This supports the early protein hypothesis in humans which states that HP intake in early childhood may increase the levels of insulin-releasing amino acids, which may consequently stimulate insulin and IGF-1 secretion and stimulate growth and adipogenic activity to increase the long-term risk of obesity and associated disorders.^30^ However, our results contradict murine studies in which HP diet resulted in reduced weight gain.^24^^,^^25^^,^^31^^,^^32^ The mice strains used in these studies are different from the BALB/c strain that we have used in the current study. A previous report showed that there are inherent differences in metabolism between different mice strains,^33^ which can explain the contradictory results.

Despite the finding that HP diet results in an increased risk of allergic sensitization, our study did not show enhancement of airway inflammation after nasal challenge as one may expect. A possible reason for this could be that our challenge protocol using high OVA dose induced maximal airway inflammation which could not be increased further. A previous study showed that intranasal challenge with increasing doses of ovalbumin resulted in a dose-dependent airway hyperresponsiveness and inflammatory cell accumulation in a mouse model of asthma.^34^ Another study in mice showed that inhalation of aerosolized *Escherichia coli* lipopolysaccharide induced a dose-dependent activation of lymphocytes and increase in airway inflammation.^35^ Consistent with our results, a recent study showed that mice fed a high fat diet had increased food allergy score without affecting the development of atopic dermatitis-like skin inflammation.^36^

It is important to highlight that our feeding protocol started at the age of 7–8 weeks. Starting the HP diet after weaning could have resulted in different results. It will also be interesting to ascertain the effect of a longer feeding protocol on IgE-mediated sensitization. Furthermore, it will also be important to determine not only the preventive but also the therapeutic effects of dietary intervention on allergic sensitization through manipulation of fat or protein content.

Further studies are needed to explain the pathophysiologic mechanisms underlying the results reported in this study. These may include, but are not limited to gastrointestinal histopathology, immunophenotyping, and analysis of gut microbiota. In addition, studying cytokines level in BALF may help better understanding of the effect of HP diet on the airway.

In conclusion, we have shown that HP diet increases the risk of allergic sensitization in mice through increased production of Th2 cytokines. Our findings should trigger both epidemiological and basic science studies in humans to better understand the association between dietary protein and atopic diseases which will hopefully be translated into clinically relevant interventions. Efforts should be made to define the upper limit of protein in the diet that does not predispose to allergic sensitization. The effect of diet on health should remain a focus of research for the establishment of optimal health and resilience.

## Abbreviations

**ND**, Normal diet; **HP**, High protein diet; **OVA**, ovalbumin; **BALF**, Bronchoalveolar lavage fluid; **H&E**, hematoxylin and eosin; **PAS**, periodic acid–Schiff; **CI**, confidence interval; **IQR**, interquartile range; **ILC2**, innate lymphoid cells; **Treg**, regulatory T cell.

## Confirmation of unpublished work

This manuscript is original, has not been published before, is not currently being considered for publication elsewhere.

## Data availability statement

The data that support the findings of this study will be available by contacting the corresponding author.

## Author contributions

WA: Development of the research concept and goals, design of methodology, funding acquisition, supervision of the research activities, data analysis, writing the initial manuscript draft and approval of the submitted manuscript. FA and RR: Design of methodology, in-vitro stimulation of splenocytes, estimation of cytokine level, data analysis, critical review of the manuscript, and approval of the submitted manuscript.

## Ethics statement

The study was approved by the Health Sciences Research Ethics Committee for Animal Studies of the College of Medicine, Kuwait University approval number 23/VDR/EC.

## Funding

This work was supported by the Kuwait Foundation for the Advancement of Science (KFAS) project number PR19-13SL-02. The funder had no role in the design, data collection, data analysis, and reporting of this study.

## Declaration of competing interest

The authors report no competing interests.
