# Supplementary material for: High protein diet increases the risk of allergic sensitization but not asthma in mice through modulation of the cytokine milieu toward Th2 bias
Source: World Allergy Organ J. 2025 Feb 6;18(2):101031. doi: 10.1016/j.waojou.2025.101031 (PMC11848459; doi:10.1016/j.waojou.2025.101031)
Supplement: Multimedia component 1 [file mmc1.docx]

**Supplementary table 1: Tests for normality of distribution and homogeneity of variance of different variables**

| **Variable** | **Normality of distribution**  **Shapiro-Wilk Test, P-value** | **Homogeneity of variance**  **Levene’s Test, P-value** |
| --- | --- | --- |
| Increase in weight | 0.3310 | 0.2652 |
| Ova-IgE | 0.2764 | 0.1403 |
| Ova-IgG1 | 0.4441 | 0.7935 |
| Ova-IgG2a | <0.0001 | 0.8551 |
| IL-17 | 0.0022 | 0.0105 |
| IFN-γ | <0.0001 | 0.2982 |
| IL-4 | 0.2168 | 0.2523 |
| IL-6 | 0.1141 | 0.6358 |
| TNF-α | 0.0470 | 0.2552 |
| Total cell count | 0.3949 | 0.4842 |
| Absolute macrophage count | 0.8169 | 0.5427 |
| Absolute lymphocyte counts | 0.6723 | 0.8234 |
| Absolute neutrophil count | 0.1458 | 0.4285 |
| Absolute eosinophil count | 0.1738 | 0.9625 |
| Mucus integrity score | 0.0360 | 0.1151 |
| Cellular infiltration score | 0.0185 | 0.3224 |
